# Online vs in-person musculoskeletal ultrasound course: a cohort comparison study

**DOI:** 10.1186/s13089-024-00375-4

**Published:** 2024-05-31

**Authors:** Shirley Lake, Ryan Brydges, Chris Penney, Diane Wilson, Raquel Sweezie, Maria Bagovich, David Bong, Susan Barr, Lynfa Stroud

**Affiliations:** 1 Division of Rheumatology, University of Toronto, Sunnybrook Health Sciences Centre, 2075 Bayview Avenue, M4N3M5 Toronto, ON Canada; 2 Department of Medicine, Unity Health Toronto, University of Toronto, Toronto, Canada; 3 Division of Rheumatology, University of Calgary, Calgary, Canada; 4 Lunenberg, Nova Scotia, Canada; 5 Sunnybrook Research Institute, Toronto, Canada; 6 University of Barcelona School of Medicine, Barcelona, Spain; 7 Department of Medicine, University of Toronto, Toronto, Canada

**Keywords:** Musculoskeletal ultrasound online education

## Abstract

**Background:**

Point-of-care musculoskeletal (MSK) ultrasound (US) courses are typically held in-person. The COVID-19 pandemic guidelines forced courses to switch to online delivery. To determine this impact, we conducted an observational cohort study, comparing homework completion and image quality between an Online and a historical In-person cohort.

**Methods:**

The In-person (*n* = 27) and Online (*n* = 24) cohorts attended two learning sessions spaced six months apart. The course content was the same, while the process of delivery differed. As homework, participants submitted US images biweekly for up to five months after each session. Expert faculty provided written feedback to all participants, and two independent reviewers rated the image quality for a subset of participants in each group who had completed at least 70% of their homework (In-person, *n* = 9; Online, *n* = 9). Participants self-reported their satisfaction through post-course evaluation.

**Results:**

63% of In-Person and 71% of Online cohort participants submitted their homework images. We observed no differences in the mean amount of homework images submitted for In-person (M = 37.3%, SD = 42.6%) and Online cohorts (M = 48.1%, SD = 38.8%; *p* > 0.05, Mann-Whitney U Test). At course end, the cohorts did not differ in overall image quality (*p* > 0.05, Wilcoxon Signed-rank Test). All participants reported high levels of satisfaction.

**Conclusions:**

A convenience sample of participants attending a basic MSK US course in-person and online did not differ statistically in homework completion, quality of submitted US images, or course satisfaction. We add to literature suggesting online learning remains a viable option post-pandemic.

**Supplementary Information:**

The online version contains supplementary material available at 10.1186/s13089-024-00375-4.

## Background

Clinicians increasingly use point of care musculoskeletal (MSK) ultrasound (US) at the bedside to aid in diagnosis and treatment of MSK diseases [1]. In 2001, the Agency for Healthcare Research and Quality listed the “use of real-time ultrasound guidance” as a key patient safety practice designed to decrease medical errors [2]. However, lack of training in MSK US is a major obstacle to its widespread use [3]. MSK US is an operator-dependent imaging modality, therefore clinicians require appropriate training to ensure skilled and safe operation. COVID-19 restrictions recently forced many courses to move their in-person MSK US training to an online format. The efficacy of a completely online longitudinal MSK US curriculum has yet to be established and requires comparison to in-person teaching.

Benefits of online learning include its cost effectiveness, reusability, convenience, and increased accessibility to excellent teachers from anywhere in the world [4]. Video conferencing can be useful to teach US scanning techniques, review US images and discuss specific point-of-care US (POCUS) topics [5–9]. The financial savings for learners and instructors include the cost and time to travel, lodging, and shipping costly US machines. Also, in pandemic circumstances, a positive is the potential safety for patients and clinicians by avoiding in-person exposure to infectious agents.

Potential downsides of online learning include time management required of learners to engage online, and the possible impact of less social presence and interaction. One review found that internet problems and difficulty communicating between students and instructors were factors that contributed to learners’ dissatisfaction with online learning compared to in-person [10]. Limited bandwidth, internet instability and different time zones can be barriers for learners [7]. Some studies demonstrate poorer outcomes from online learning among certain groups of students (such as those based on race, ethnicity, ability, sex, etc.) [4]. Additionally, faculty express less satisfaction during tele-ultrasound scanning regarding their ability to engage learners, troubleshoot image acquisition, and provide feedback [11].

Most previous studies comparing didactic vs. hands-on training in POCUS show that hands-on training with or without didactic training had better outcomes in terms of knowledge, image acquisition, image interpretation, confidence, and procedural skills than didactic alone [12]. Hence, hands-on practice appears to be critical in developing competence in POCUS. Some recommend that 50% of a workshop or course in ultrasound be devoted to supervised, hands-on training [13]. When comparing web-based and in-person didactic POCUS training, a review of several studies showed no difference in knowledge, image acquisition, image interpretation, procedural skills, clinical decision making, and confidence [12]. Additional studies of online courses replacing in-person hands-on courses for POCUS training have shown learner and educator satisfaction, confidence, and effectiveness in acquiring and interpreting US images [5, 14]. Recent studies have shown no difference in POCUS knowledge [6, 15], including one study that compared imaging skills and quality of US images from an in-person vs. online taught cohort through evaluation of US images submitted asynchronously [16].

In the present study, we aimed to determine whether learning outcomes differed among participants in the in-person and online formats of an established basic MSK US course. Specifically, we compared homework completion rates and quality of images submitted longitudinally by participants enrolled in the historical pre-pandemic (in-person) and post-pandemic (online) courses. We also compared participants’ self-reported satisfaction with each course’s content and structure.

## Materials and methods

### Study setting

The Canadian Rheumatology Ultrasound Society (CRUS) has delivered a basic MSK POCUS course annually since 2010. Originally designed as an in-person blend of didactic and hands-on learning, the course takes place over two weekend sessions spaced five months apart. After each session, participants practice their MSK US technique, and receive expert feedback by submitting US images online every two weeks for at least three months. Due to the COVID-19 pandemic, the course switched to a fully online didactic format.

### Study design and participants

We conducted an observational cohort comparison trial, comparing a historical cohort from 2018 to 2019 that completed the in-person format of the MSK US course (“In-person cohort”) to a 2020–2021 cohort that completed the online format (“Online cohort”).

Participants were recruited from registrants for the CRUS basic MSK US course. All course participants were emailed an information leaflet and consent form approved by the Sunnybrook Health Sciences ethics committee for voluntary participation (REB 404–2019). Anyone who declined to participate was not included in the study. Participants included rheumatologists and physicians in physical medicine and rehabilitation, and trainees within these disciplines.

### Course descriptions

Each course followed the same 8-month curriculum including two weekend sessions, one in October and another in March. During each weekend session, nine expert faculty mentors demonstrated how to obtain standard scans and reviewed basic pathology for specific rheumatic diseases. Scanning techniques included the acquisition of power Doppler and greyscale images in both transverse and longitudinal views. The first weekend focused on scanning small joints (*In-person*: 5.5 h didactic, 6.5 h hands-on; *Online*: 7 h didactic, 0 h hands-on) and the second weekend focused on scanning large joints (*In-person*: 5.5 h didactic, 6.5 h hands-on; *Online*: 8 h didactic, 0 h hands-on). More didactic time in the Online course was dedicated to observed US demonstrations and interactivity such as question and answer periods. Scanning techniques were taught for each anatomical area with a focus on a specific standard scans of the small joints (i.e., dorsal metacarpal phalangeal (MCP), dorsal proximal interphalangeal (PIP), dorsal metatarsal phalangeal (MTP), dorsal wrist, volar wrist, and anterior tibiotalar joint), and the large joints (shoulder biceps, shoulder supraspinatus, elbow humeroradial, elbow lateral tendon, and knee suprapatellar).

Participants submitted homework (i.e., deidentified US images of standard scans) electronically in batches every two weeks for either 16 weeks (In-person, 8 batches) or 14 weeks (Online, 7 batches) after the Small Joints session, and 12 weeks after the Large Joints session (6 batches each). The total maximum possible number of batches participants could submit for the whole course was fourteen for the In-person cohort (8 + 6 batches) and thirteen for the Online cohorts (7 + 6 batches). Each batch of homework included standard greyscale and power Doppler scans of the anatomical areas, and transverse and longitudinal scans for all joint areas except the elbow (humeroradial and lateral tendon) where only longitudinal views were required. Therefore, per batch, participants submitted two to four scans for each anatomical area. In total, participants were invited to submit a maximum of 192 images of the small joints session, and a maximum of 96 images of the large joints.

All batches of homework in both groups were deidentified and randomly assigned to different mentors for biweekly feedback. Mentors reviewed images and commented on improving participants’ positioning of the probe (rotation, angulation, pressure and placement over the correct bony landmarks), and adjustment of machine settings (depth, frequency, focal zone, gain and pulse repetition frequency). As part of the online instructional approach, participants could also attend online coaching sessions with assigned mentors. The coaches were not the same individuals who reviewed homework batches.

### Learning outcomes

Two independent, blinded raters used published criteria [15] to rate the image quality of all scans from participants who completed greater than 70% of their homework in both groups. Image quality was globally graded on a scale from 1 to 5 (1 = poor and 5 = publication quality) with scans achieving a score of *≥* 3 considered acceptable (see Supplementary Table S1 for US evaluation tool). Grading emphasized the following image qualities: clarity of bone contour, avoidance of anisotropy, transducer positioning, pressure and orientation relative to structures of interest, and equipment settings. For each anatomical area, we calculated an overall image quality score for each batch by averaging the two raters’ scores. Raters scored four batches of homework: the first and final batches submitted after each session (Small Joints batches 1 and 8, Large Joints batches 1 and 6).

Course satisfaction was measured through an end of course evaluation form (see Supplementary Figure S1). Participants rated whether the program met their expectations on a 5-point Likert scale from very poor to excellent in the In-person cohort, and from strongly disagree to strongly agree in the Online cohort.

### Data analysis

We performed all analyses using SPSS version 25 (IBM, Armonk, NY). We analyzed homework completion using Mann-Whitney U tests and Chi-square tests to compare the In-person and Online cohort. For image quality, we compared scores between batches submitted in the first and final weeks for each anatomical area using a Wilcoxon signed rank test. We first compared scores for the specific anatomical areas (e.g., tibiotalar vs. dorsal MCP) using Friedman’s test, and found image quality did not vary with anatomical area for small vs. large joints, nor between cohorts (see Supplementary Table S2). Consequently, we pooled scores into an overall average quality score for small vs. large joints for all remaining analyses. We used Mann-Whitney U tests to compare the overall image quality between In-person and Online cohorts for batches submitted in the first and final weeks. All *p* values for Mann Whitney and Wilcoxon signed rank tests were adjusted using a Bonferroni correction when two or more variables were being compared. P- values < 0.05 are considered significant.

Finally, we compared the number of students who improved in overall image quality from first to final week for each specific joint component. A positive value indicated an improvement in overall image quality. For each anatomical area, the number of participants demonstrating an improvement in overall image quality from the first to final week were compared between In-person and Online using Fisher’s Exact test. We collected data on two convenience samples and did not conduct a prospective power or sample size calculation given we had no control over the number of participants enrolled in the course.

## Results

### Homework completion

We had 27 students enrolled in the In-person cohort, and 24 students enroll in the Online cohort (Table 1). We observed similar homework completion rates in the In-person and Online cohorts (Table 1). Of the maximum number of possible homework batches, the mean (SD) percentage completed by participants in the In-person 37.3% (42.6) and Online cohorts 48.1% (38.8) did not differ statistically (*p* = 0.475). The uptake of contacting online mentors was infrequent, with less than 50% of Online students (four students) utilizing this opportunity.

**Table 1:** Participant homework completion

	In-person Cohort	Online Cohort	*P* values
Enrollment	27	24	--
Number of students who submitted US homework [n (%)]	17 (63)	17 (71)	0.55^a^
Batches completed per student, Mean (SD) [Total Batches completed/Total Batches Assigned (%)]	37.3 (42.60)	48.1 (38.84)	0.47^b^
Number of students who completed all US homework batches [n (%)]	5 (19)	4 (17)	0.86^a^
Number of students who completed < 2 US homework batches [n (%)]	15(56)	7 (29)	0.17^a^
Number of students who completed > 70% of US homework batches [n (%)]	9 (33)	9 (38)	1.00^a^

^a^Chi-square test, α = 0.05; ^b^Mann-Whitney U Test, α = 0.05

### Overall image quality

Image quality did not vary with each anatomical area, nor between cohorts (Supplementary Table S2). Notably, the inter-rater reliability between the two raters was low (ICC < 0.50), whether the scores were lumped together or by each separate joint area (Supplementary Table S3). We chose to analyze the overall average quality score for all small vs. all large joints for all remaining analyses. We observed that both cohorts improved from the first to final week following the Small Joints session (*p* < 0.018 and 0.010), but not the Large Joints session (*p* > 0.05; Table 2). The overall image quality did not differ between the In-person and Online cohorts before or after either the small or large joints sessions (Table 2).

**Table 2:** Pooled overall image quality scores: comparison of in-person and online cohorts for small and large joints

		In-Person		Online		*p*	Adjusted p
Course		n	Median (IQR)	n	Median (IQR)		
**Small Joints**	First Week Overall Image Quality	54	2.75 (0.53)	42	2.63 (0.75)	0.111	0.333
	Final Week Overall Image Quality	54	3.00 (0.38)*	54	2.88 (0.50)*	0.221	0.663
	Δ Overall Image Quality	54	0.13 (0.63)	42	0.19 (0.75)	0.500	1.000
**Large Joints**	First Week Overall Image Quality	40	2.88 (0.75)	35	2.75 (0.63)	0.605	1.000
	Final Week Overall Image Quality	35	3.00 (1.00)	25	3.00 (0.63)	0.689	1.000
	Δ Overall Image Quality	30	0.00 (0.53)	20	0.13 (0.81)	0.474	1.000

* Significant improvement in overall small joint image quality in final week compared to first week for both the In-person (*p* = 0.009; adjusted *p* = 0.018) and the Online (*p* = 0.005; adjusted *p* = 0.010) cohorts.

### Participant improvement comparison

The number of participants demonstrating improvement in overall image quality is shown in Table 3. There were no appreciable differences between the two cohorts in the number of participants with a positive change in overall image quality for each of the anatomical areas.

**Table 3:** Number of participants demonstrating improvement in overall image quality

Session	Anatomical Area	In-person				Online				*p*
		Improvement		No Improvement		Improvement		No Improvement		
		n	%	n	%	n	%	n	%	
**Small Joints**	Dorsal MCP	8	89	1	11	3	43	4	57	0.11
	Dorsal PIP	5	56	4	44	5	71	2	29	0.63
	Dorsal MTP	7	78	2	22	5	71	2	29	1.00
	Tibiotalar	2	22	7	78	5	71	2	29	0.13
	Dorsal Wrist	6	67	3	33	2	29	5	71	0.31
	Volar Wrist	6	67	3	33	4	57	3	43	1.00
**Large Joints**	Elbow Humeroradial	2	33	4	67	2	50	2	50	1.00
	Elbow Lateral Tendon	1	17	5	83	1	25	3	75	1.00
	Shoulder Biceps Tendon	3	50	3	50	3	75	1	25	0.57
	Shoulder Supraspinatus	2	33	4	67	2	50	2	50	1.00
	Knee Suprapatellar	2	33	4	67	3	75	1	25	0.52

Comparison of In-person and Online cohorts. *P*-values < 0.05 are considered significant. Nine participants in the In-person and 7 in the Online cohort completed both the first and final batches following the Small Joints session. Six participants in the In-person and 4 in the Online cohort completed both the first and final batches following the Large Joints session.

### Course satisfaction

The post-course evaluation forms revealed high satisfaction scores that were similar in both groups. In the In-person cohort, 10 of 27 people completed the post-course evaluation form. Five people stated the course content was excellent and the other five rated it very good. In the Online cohort, everyone completed the evaluation, and all students either strongly agreed (88%) or agreed (12%) that the course met their expectations.

## Discussion

Our observational cohort comparison of an in-person basic MSK US course and an online course evaluated participants’ homework completion and skill acquisition as primary outcomes. In our convenience sample of participants, we did not find any differences in their homework completion rates, nor in their acquisition of US skills measured using an overall image quality score. Participants in both cohorts expressed a high level of satisfaction. Most learners improved in their US skills over time. When pooled together, both the In-person and Online cohorts improved in small joint image quality scores from the first to final weeks.

Similar MSK US courses have been developed, but this is the first study to evaluate MSK US competency by completely online means. Our findings align with previous studies showing no difference in outcomes between online and in-person ultrasound training [6, 11, 16]. Hence, our study appears to add evidence to a perspective that basic US image acquisition may not depend on in-person contact between instructor and learner, and can be achieved through remote learning. This method has the advantage of providing students with the autonomy to obtain US images on their own schedules.

Our study found a high level of satisfaction experienced by the learners in the Online course, like other studies [5–9]. Online learning may have provided greater flexibility in scheduling or provided opportunity to those geographically challenged to participate in person [7, 16]. It is unclear whether the impact of the pandemic on normal activities may have enabled participants to dedicate more time to US homework. The option of online mentorship, although not frequently utilized, may have also encouraged greater US homework completion. In both the In-person and Online groups, about a third of students did not submit any homework, while another third completed > 70% of the homework and were responsible for most of the batches submitted. Our overall homework submission rate was around 40–50%, which is similar to other studies that had 50% and 30% completion rate [17, 18]. Completing the US homework is a key component of learning MSK US as it requires continued practice.

Our study had several limitations. While we found that US image quality for small joints pooled together demonstrated some improvement over the course, image quality for large joints and individual anatomical areas did not show statistically significant improvement likely due to our small convenience sample sizes, which resulted in underpowered analyses. Our analyses are also limited by the poor inter-rater reliability, which we attribute to the nature of our pragmatic study design; notably, the course directors are aiming to address this issue via rater training and standardization of scoring processes. Multiple cohorts would need to be studied to achieve sufficient prospective power in future related studies [19]. Our pooled scores only included participants who submitted > 70% of ultrasound homework images, and our results may be biased by these motivated learners. We used a historical cohort that introduces confounders such as the lack of randomization and blinding, leading to potential performance, detection, and attrition bias. The amount of didactic learning differed between the two groups, owing to the demonstration of US techniques in the online cohort. The course satisfaction outcomes were captured using different scales, and fewer than 50% completed the post-course evaluation in the historical cohort, which may limit the generalizability of our satisfaction results. Not all learners in the Online cohort utilized the virtual mentorship, and we were unable to survey participants to understand the reasons behind this. We also did not capture faculty satisfaction. Faculty perspectives would provide valuable insights into the challenges of online mentorship and teaching in future studies of MSK US education. We also suggest that the short 8-month course likely limited participants’ capacity to significantly improve their skills. Indeed, US skills continue to develop with ongoing practice and experience, and longer curricula lead to better image acquisition, image interpretation, and knowledge retention [12].

Our study also had several strengths. We assessed US competency based on submitted US images, which is a feasible and practical way to assess US skills. This contrasts with studies that evaluate students using multiple-choice tests [11, 16] or by self-assessment [5, 6, 14]. Our choice to use two independent, blinded reviewers provides a rigorous way to rate skills and collect robust data. The US images were also evaluated over time, months after the didactic/hands-on teaching, thus demonstrating learning retention. Our study also shows that learners can acquire basic US skills even without dedicated local mentors. Online learning of MSK US appears to be a viable option that warrants further research and development.

## Conclusion

In summary, our study showed that completely online learning of basic MSK US is both feasible and appreciated, especially when there are logistical concerns related to meeting in-person. As such, learners’ demands for remote and hybrid online options for learning is something organizers must continue to consider when planning future courses.

### Electronic supplementary material

Below is the link to the electronic supplementary material.

Supplementary Material 1

Supplementary Material 2

## Data Availability

All data generated or analysed during this study are included in this published article and its supplementary information files.
