# Supplementary material for: Online vs in-person musculoskeletal ultrasound course: a cohort comparison study
Source: Ultrasound J. 2024 May 31;16:30. doi: 10.1186/s13089-024-00375-4 (PMC11143147; doi:10.1186/s13089-024-00375-4)
Supplement: Supplementary file 1 — Supplementary Material 1 [file 13089_2024_375_MOESM1_ESM.docx]

**
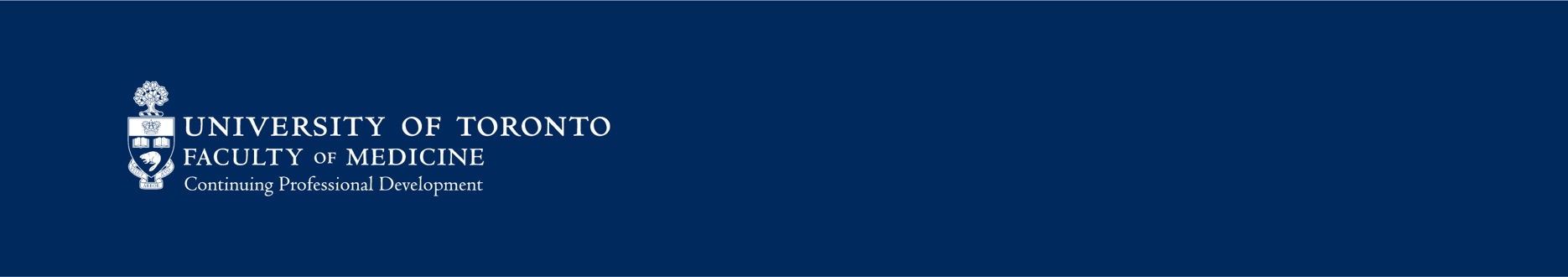
**

Canadian Rheumatology Ultrasound Society Basic Course

October 19-20^th^, 2019 and March 28-29^th^, 2020

**PART 1: Program/Session Evaluation** ^[[1]](#footnote-1)^

Please rate the following aspects of the program/conference:

|  | **1** | **2** | **3** | **4** | **5** |
| --- | --- | --- | --- | --- | --- |
|  | Strongly Disagree | Disagree | Neutral | Agree | Strongly Agree |
| The program/session met the stated learning objectives |  |  |  |  |  |
| 1.Describe the application, indication and limitations of MSK US in rheumatology |  |  |  |  |  |
| 2. Identify sonographic patterns of different MSK tissues and recognize MSK artifacts and pitfalls in obtaining optimal MSK US images |  |  |  |  |  |
| 3. Demonstrate the correct method of holding the US probe, and locate and recognize normal anatomy and pathology of the finger, wrist, elbow, shoulder, hip, knee, ankle and feet joints |  |  |  |  |  |
| 4. Build- on MSK US skills to consider further training and certification in MSKUS in rheumatology |  |  |  |  |  |
| The program/session content … | | | | | |
| Was relevant to my discipline/profession |  |  |  |  |  |
| Met my expectations |  |  |  |  |  |
| Was well organized |  |  |  |  |  |
| Disclosure of potential conflicts of interest was clearly communicated |  |  |  |  |  |
| There were adequate opportunities to interact with my peers |  |  |  |  |  |
| I will use the information I learned in my practice |  |  |  |  |  |

***Did you perceive any degree of bias in any part of the program?**

- Yes
- No

*If yes, please describe: ________________________________________________________________

**What was the most effective part of the program/session? Why?**

**____________________________________________________________________________________**

***The program/session will encourage me to consider changes in my current practice**

- YES: The changes I am considering are: _____________________________________________________________________________
- NO: because:
- I do not see the need to make changes to my current practice.
- I am thinking about changes and have the necessary resources but not ready to make them yet
- I am already implementing recommended practices
- The information was not relevant to me
- I do not have the required resources to implement these changes

**What suggestions do you have for improving the program/session?**

**____________________________________________________________________________________**

**Please list any topics you would like to see addressed in future program/sessions:**

**____________________________________________________________________________________**

***For physicians only: please indicate which CanMEDS/CanMEDS-FM roles you felt were addressed during this program/session?** (this is a required question for physicians only)

- Medical Expert/Family Medicine Expert
- Scholar
- Collaborator
- Communicator
- Leader
- Professional
- Health Advocate

**PART 2: Session/Speaker(s) Evaluation**

| **Please rate the quality of the session/speaker(s) on a scale of 1 (poor) to 5 (excellent)** | | | | | | | | | | | | | | | | | | | | | | | | | |
| --- | --- | --- | --- | --- | --- | --- | --- | --- | --- | --- | --- | --- | --- | --- | --- | --- | --- | --- | --- | --- | --- | --- | --- | --- | --- |
|  | Met Stated Objectives | | | | | Program Content Enhanced My Knowledge | | | | | Balanced ^[[2]](#footnote-2)^ and Unbiased | | | | | Relevance to Practice Overall | | | | | Time for Active Learning | | | | |
| **Saturday** |  | | | | |  | | | | |  | | | | |  | | | | |  | | | | |
| Basic physics of US -Lake | 1 | 2 | 3 | 4 | 5 | 1 | 2 | 3 | 4 | 5 | 1 | 2 | 3 | 4 | 5 | 1 | 2 | 3 | 4 | 5 | 1 | 2 | 3 | 4 | 5 |
|  |  |  |  |  |  |  |  |  |  |  |  |  |  |  |  |  |  |  |  |  |  |  |  |  |  |
| Anatomy – Agur | 1 | 2 | 3 | 4 | 5 | 1 | 2 | 3 | 4 | 5 | 1 | 2 | 3 | 4 | 5 | 1 | 2 | 3 | 4 | 5 | 1 | 2 | 3 | 4 | 5 |
|  |  |  |  |  |  |  |  |  |  |  |  |  |  |  |  |  |  |  |  |  |  |  |  |  |  |
| Hands and Wrist – Larche | 1 | 2 | 3 | 4 | 5 | 1 | 2 | 3 | 4 | 5 | 1 | 2 | 3 | 4 | 5 | 1 | 2 | 3 | 4 | 5 | 1 | 2 | 3 | 4 | 5 |
|  |  |  |  |  |  |  |  |  |  |  |  |  |  |  |  |  |  |  |  |  |  |  |  |  |  |
| Hand and wrist pathology - Eder | 1 | 2 | 3 | 4 | 5 | 1 | 2 | 3 | 4 | 5 | 1 | 2 | 3 | 4 | 5 | 1 | 2 | 3 | 4 | 5 | 1 | 2 | 3 | 4 | 5 |
| **Sunday** |  | | | | |  | | | | |  | | | | |  | | | | |  | | | | |
|  |  |  |  |  |  |  |  |  |  |  |  |  |  |  |  |  |  |  |  |  |  |  |  |  |  |
| Foot and ankle – Bagovich | 1 | 2 | 3 | 4 | 5 | 1 | 2 | 3 | 4 | 5 | 1 | 2 | 3 | 4 | 5 | 1 | 2 | 3 | 4 | 5 | 1 | 2 | 3 | 4 | 5 |
|  |  |  |  |  |  |  |  |  |  |  |  |  |  |  |  |  |  |  |  |  |  |  |  |  |  |
| Foot pathology –Wilson | 1 | 2 | 3 | 4 | 5 | 1 | 2 | 3 | 4 | 5 | 1 | 2 | 3 | 4 | 5 | 1 | 2 | 3 | 4 | 5 | 1 | 2 | 3 | 4 | 5 |

Additional comments related to the session/speaker(s):

**____________________________________________________________________________________**

**PART 3: Program / Conference Logistics**

|  | **1** | **2** | **3** | **4** | **5** | **N/A** |
| --- | --- | --- | --- | --- | --- | --- |
|  | Strongly Disagree | Disagree | Neutral | Agree | Strongly Agree | Not Applicable |
| The registration process was easy to use |  |  |  |  |  |  |
| Information about the program on the website was useful |  |  |  |  |  |  |

Additional comments related to the Program/Conference Logistics:

**____________________________________________________________________________**

**PART 4: About You**

**What attracted you to attending this program/conference (Please select all that apply).**

- Updating Knowledge
- Wanted to change how I do things in my practice
- Speaker(s)
- Networking
- Location
- Accredited Program

**What is your health profession, role, discipline?** **Please check all that apply.**

- Family Physician
- Rheumatologist
- Specialist: ______________
- Resident : ______________
- Medical Student
- Physician Assistant
- Health professional (not listed above)
- N/A
- Other (Please describe)___________

**How many years have you been in practice?**

- Not in practice
- < 10 years
- 10-20 years
- 20-30 years
- 30+ years
- N/A

1. Questions with an asterisk in front of them are required for CFPC and/or RCPSC accreditation. Please do not remove or reword these questions. [↑](#footnote-ref-1)
2. By ‘balanced’, we mean that all options and points of view are given impartially with appropriate evidence. [↑](#footnote-ref-2)
