# Supplementary material for: Online vs in-person musculoskeletal ultrasound course: a cohort comparison study
Source: Ultrasound J. 2024 May 31;16:30. doi: 10.1186/s13089-024-00375-4 (PMC11143147; doi:10.1186/s13089-024-00375-4)
Supplement: Supplementary file 2 — Supplementary Material 2 [file 13089_2024_375_MOESM2_ESM.docx]

**Supplementary Material**

**Table S1**: US Evaluation Tool from Kissin et al (15).

| **Score** | **Category** | **Descriptor** |
| --- | --- | --- |
| **0** |  | No image |
| **1** | Unacceptable | Image not adequate to draw conclusions. Eg not all structures seen, anisotropy, lack of bony contour, settings wrong |
| **2** | Below acceptable | Image barely adequate for drawing clinical conclusions. 1 major fault eg. incorrect settings, lack bony contours, missing structure |
| **3** | Acceptable | Adequate, average image. All structure seen. 1 or 2 minor fault eg. Bony cortex could be sharper, doppler box bigger, small artifact, less pressure/ gel layer |
| **4** | Very Good | Adequate, good image. Bony contour sharp, avoid anisotropy, transducer positioning correct and all structures seen, not too much pressure, equipment setting correct |
| **5** | Excellent | Adequate, Publication quality image |

**Table S2**: Overall Image Quality: Comparison of first week vs final week for each joint in each cohort. Significance is for p < 0.05 (Wilcoxon signed rank test, p value adjusted with a Bonferroni Correction).

|  | | **In-Person** | | | | | | **Virtual** | | | | |  |
| --- | --- | --- | --- | --- | --- | --- | --- | --- | --- | --- | --- | --- | --- |
|  |  | **First Week** | | **Final Week** | | **p** | **Adjusted p** | **First Week** | | **Final Week** | | **p** | **Adjusted p** |
| **Course** | **Joint Component** | **n** | **Median (IQR)** | **n** | **Median (IQR)** |  |  | **n** | **Median (IQR)** | **n** | **Median (IQR)** |  |  |
| **Small Joints** | Dorsal MCP | 9 | 2.75 (0.62) | 9 | 3.25 (0.38) | 0.010 | 0.061 | 7 | 2.75 (0.50) | 9 | 3.12 (0.50) | 0.399 | 1.000 |
|  | Dorsal PIP | 9 | 3.00 (0.62) | 9 | 3.00 (0.31) | 0.287 | 1.000 | 7 | 2.75 (0.75) | 9 | 3.25 (1.00) | 0.141 | 0.847 |
|  | Dorsal MTP | 9 | 2.63 (0.69) | 9 | 3.00 (0.16) | 0.171 | 1.000 | 7 | 2.50 (0.38) | 9 | 2.88 (0.44) | 0.233 | 1.000 |
|  | Tibiotalar | 9 | 3.00 (0.25) | 9 | 2.88 (0.44) | 0.209 | 1.000 | 7 | 2.50 (1.25) | 9 | 2.88 (0.50) | 0.058 | 0.351 |
|  | Dorsal Wrist | 9 | 2.75 (0.38) | 9 | 2.75 (0.44) | 0.472 | 1.000 | 7 | 2.88 (0.75) | 9 | 2.88 (0.62) | 0.892 | 1.000 |
|  | Volar Wrist | 9 | 2.75 (0.65) | 9 | 2.75 (0.69) | 0.473 | 1.000 | 7 | 2.50 (1.38) | 9 | 2.75 (0.44) | 0.141 | 0.847 |
| **Large Joints** | Elbow Humeroradial | 8 | 2.88 (0.69) | 7 | 3.50 (0.50) | 0.581 | 1.000 | 7 | 2.50 (0.75) | 5 | 3.00 (0.75) | 0.785 | 1.000 |
|  | Elbow Lateral Tendon | 8 | 2.75 (1.50) | 7 | 3.00 (2.00) | 0.461 | 1.000 | 7 | 2.75 (0.75) | 5 | 3.00 (1.38) | 0.414 | 1.000 |
|  | Shoulder Biceps Tendon | 8 | 3.00 (0.68) | 7 | 2.62 (0.62) | 0.528 | 1.000 | 7 | 2.75 (0.38) | 5 | 2.88 (0.44) | 0.141 | 0.704 |
|  | Shoulder Supraspinatus | 8 | 3.00 (1.25) | 7 | 3.00 (1.62) | 0.416 | 1.000 | 7 | 3.00 (1.38) | 5 | 3.12 (1.06) | 0.461 | 1.000 |
|  | Knee Suprapatellar | 8 | 2.69 (0.97) | 7 | 2.88 (0.50) | 0.786 | 1.000 | 7 | 2.75 (0.71) | 5 | 2.88 (0.88) | 0.465 | 1.000 |

**Table S3**: Inter-rater reliability for two experts. N represents the number of ratings given by each of two expert raters. ICC = intraclass correlation coefficient, two-way mixed model, absolute agreement, average measures. * p < 0.05. Reliability was defined as poor (ICC < 0.50), moderate (0.50 ≤ ICC < 0.75), good (0.75 ≤ ICC < 0.90), or excellent (ICC ≥ 0.90) (ref below).

| **Scores** | **N** | **ICC** | **Upper Bound** | **Lower Bound** | **Significance** |
| --- | --- | --- | --- | --- | --- |
| All Average | 339 | 0.162 | 0.338 | -0.058 | 0.003* |
| All Doppler Transverse | 285 | 0.158 | 0.322 | -0.041 | 0.022* |
| All Grey Scale Transverse | 285 | 0.137 | 0.299 | -0.055 | 0.037* |
| All Doppler Longitudinal | 339 | 0.195 | 0.352 | 0.000 | 0.005* |
| All Grey Scale Longitudinal | 339 | 0.167 | 0.342 | -0.052 | 0.003* |
| Small Joints Average | 204 | 0.274 | -0.080 | 0.507 | 0.000* |
| Large Joints Average | 135 | -0.26 | -0.242 | 0.175 | 0.599 |
| MCP Dorsal Average | 34 | 0.117 | -0.301 | 0.463 | 0.280 |
| PIP Dorsal Average | 34 | 0.500 | -0.064 | 0.762 | 0.003* |
| MTP Dorsal Average | 34 | 0.091 | -0.264 | 0.418 | 0.303 |
| Wrist Dorsal Average | 34 | 0.057 | -0.252 | 0.369 | 0.363 |
| Wrist Volar Average | 34 | 0.247 | -0.212 | 0.573 | 0.091 |
| Tibiotalar Average | 34 | 0.362 | -0.178 | 0.673 | 0.019* |
| Shoulder Biceps Tendon Average | 27 | 0.045 | -0.288 | 0.390 | 0.402 |
| Should Supraspinatus Average | 27 | -0.064 | -0.667 | 0.407 | 0.594 |
| Elbow Humeroradial Average | 27 | 0.324 | -0.223 | 0.668 | 0.026* |
| Elbow Lateral Tendon Insertion Average | 27 | -0.151 | -0.782 | 0.353 | 0.717 |
| Knee Suprapatellar | 27 | -0.151 | -0.772 | 0.350 | 0.720 |

Koo TK, Li MY**.** A Guideline of Selecting and Reporting Intraclass Correlation Coefficients for Reliability Research. J Chiropr Med. 2016;15(2):155-63.
